# HLA-G and the MHC Cusp Theory

**DOI:** 10.3389/fimmu.2022.814967

**Published:** 2022-02-25

**Authors:** Bruna Miglioranza Scavuzzi, Vincent van Drongelen, Joseph Holoshitz

**Affiliations:** Department of Internal Medicine, University of Michigan, Ann Arbor, MI, United States

**Keywords:** human leukocyte antigen (HLA), HLA-G, Cusp theory, antigen presentation (AP), HLA-disease association, immunity

## Abstract

Human leukocyte antigens (HLA) are significant genetic risk factors in a long list of diseases. However, the mechanisms underlying these associations remain elusive in many cases. The best-characterized function of classical major histocompatibility complex (MHC) antigens is to allow safe presentation of antigenic peptides *via* a self/non-self-discrimination process. Therefore, most hypotheses to date have posited that the observed associations between certain HLA molecules and human diseases involve antigen presentation (AP). However, these hypotheses often represent inconsistencies with current knowledge. To offer answers to the inconsistencies, a decade ago we have invoked the MHC Cusp theory, postulating that in addition to its main role in AP, the MHC codes for allele-specific molecules that act as ligands in a conformationally-conserved cusp-like fold, which upon interaction with cognate receptors can trigger MHC-associated diseases. In the ensuing years, we have provided empirical evidence that substantiates the theory in several HLA-Class II-associated autoimmune diseases. Notably, in a recent study we have demonstrated that *HLA-DRB1* alleles known to protect against several autoimmune diseases encode a protective epitope at the cusp region, which activates anti-inflammatory signaling leading to transcriptional and functional modulatory effects. Relevant to the topic of this session, cusp ligands demonstrate several similarities to the functional effects of HLA-G. The overall goal of this opinion article is to delineate the parallels and distinctive features of the MHC Cusp theory with structural and functional aspects of HLA-G molecules.

## Introduction

The major histocompatibility complex (MHC), known in humans as HLA (human leukocyte antigen) is a cluster of genes nested in three regions, designated as Class I, Class II and Class III. Class I and II genes encode for molecules best known for their role in presentation of antigenic peptides through a self/non-self-discrimination process (1). Paradoxically, however, in spite of this meticulous self-protecting mechanism, particular HLA alleles and haplotypes are some of the most significant genetic risk factors in numerous human diseases (2). Over the decades, several hypotheses have been put forward to explain the mechanistic basis of the associations. Most of the salient hypotheses are based on the best-known function of HLA molecules: antigen presentation (AP). Among them: (*i*) Molecular mimicry with foreign antigens (3). (*ii*) Failures in T cell repertoire selection (4); (*iii*) Reactivity to altered self-antigens (5); (*iv*) Association through linkage disequilibrium (LD) (6). Although progress has been made (7), most hypotheses have not been yet empirically validated, and AP flaws as a sole cause are incongruent with current knowledge, as we previously discussed (8, 9).

Given the limitations of AP-based hypotheses, a decade ago we have proposed an alternative theory (8, 9), postulating that in addition to their main role in AP, MHC molecules also express allelic epitopes, which activate immune modulatory pathways through interactions with cell surface receptors. Depending on permissive background genes and environmental influences, these otherwise physiologic pathways can excessively activate cellular events that lead to disease onset. We branded the new theory ‘MHC Cusp’ based on our prior finding that an epitope coded by a rheumatoid arthritis (RA)-associated *HLA-DR1* third allelic hypervariable region acts as a signaling ligand that can cause immune dysregulation and lead to autoimmune arthritis in experimental mouse models (10–23). The term ‘cusp’ was chosen based on the conformational properties of the epitope, which is located near a cusp-like prominence that is a common MHC fold feature shared by antigen presenting, as well as non-antigen presenting members of the MHC family. Since its postulation, we have provided empirical evidence in support of the MHC Cusp theory in RA, and more recently in two other diseases associated *DRB1* alleles. Directly relevant to the focus of this review, we have recently identified a new cusp ligand that is encoded by RA-protective *DRB1* alleles. That allelic cusp region, which we designated as protective epitope (PE), has the amino acid sequence DERAA in the cusp region (residues 70-74 of the DRβ chain), and activated anti-inflammatory transcriptomes as well as signaling events that culminate in immune modulatory effects (23). Being an HLA ligand that activates immune tolerance effect, the PE demonstrates resemblance to some of the widely documented HLA-G effects.

In the following segments, we review structural and functional aspects of classical versus the non-classical HLA molecules, discuss salient mechanistic theories of HLA-disease association, expend on the MHC Cusp theory and its parallels, as well as distinguishing features with functional and structural features of HLA-G ligands, and potential of harnessing these parallels for future exploration of new therapeutic strategies.

## HLA Molecules in Health and Disease

### Structure and Canonical Functions of HLA Molecules

The HLA complex is a cluster of genes located on the short arm of chromosome 6 p21.3, that encode for MHC molecules in humans (24). The HLA is one of the most polymorphic segments in the human genome, having more than 45 genes accounting for over 25,000 currently known alleles (25). The HLA consists of three major regions, termed class I, containing classical (Ia) genes (*HLA-A, -B, -C*), as well as the nonclassical HLA (Ib) genes (*HLA-E, -F, -G, -H*), The HLA class II region contains the classical class II genes (*HLA-DRA, -DRB1, -DRB2, -DRB3, -DRB4, -DRB5, -DQA1, -DQA2, -DQB1, -DQB2, -DPA1, -DPB1*) and other, less variable, genes (*HLA-DMA, -DMB, -DOA, -DOB*). The class III region contains complement factor genes (*e.g. C2*, *C4*) and other genes related to inflammation, leukocyte maturation and immune response (*e.g. TNF, HSPA1*) (26).

HLA class I and II genes encode for transmembrane glycoproteins best known for their role in presentation of antigenic peptides (1). The MHC class I molecule is typically composed of one α heavy chain, containing two peptide-binding domains (α1, α2), one immunoglobulin-like domain (α3), a transmembrane region and a cytoplasmic tail, as well as a light chain, a non-HLA encoded low molecular protein, named Beta-2 microglobulin (β_2_m) (27). The MHC class II molecule is composed of two heavy chains (α, β), each containing: a peptide-binding domain (α1 and β1, respectively), immunoglobulin-like domain (α2 and β2, respectively), a transmembrane region and a cytoplasmic tail (27). Three-dimensional structures of MHC Class I and II molecules determined by x-ray crystallography revealed a similar fold and peptide-binding groove between the two classes of molecules (28, 29).

MHC Class I molecules are expressed on the cell surface of human nucleated cells and typically present short endogenous peptides to cytotoxic CD8 T cells (30, 31). MHC class I molecules may also present exogenous peptides, in a process called “cross-presentation” (32). MHC class II molecules are expressed on the surface of professional antigen presenting cells (APCs), such as macrophages, dendritic cells, and B cells. They typically present exogenously synthesized antigens to CD4 T cells (33, 34).

HLA-G molecules are best known for their immunomodulatory properties and their involvement in maternal–foetal and organ transplantation immune tolerance (35). They act as ligands and have the ability to bind to inhibitory receptors expressed by a variety of immune cells (36). HLA-G molecules exert inhibitory effects on both innate and adaptative immune responses, such as inhibition of natural killer (NK) cells, as well modulation of B and T cell function (37). Their interaction with immunoglobulin-like receptors, such as receptor Ig-like transcripts 2 and 4 (ILT2, ILT4) and killer cell immunoglobulin-like receptor (KIR) KIR2DL4 expressed by peripheral NK cells, B- and T lymphocytes and monocytes, leading to inhibitory function has been well described (36, 38). HLA-G has also been shown to increase apoptosis of activated CD8^+^ T cells and NK cells, as well as to reduce chemotaxis by downregulation of chemokine receptor expression in T cells (36). Moreover, its expression can be transactivated by the *AIRE* (Autoimmune Regulator) gene, the main regulator of negative selection in the thymus, which suggests a possible involvement of this molecule in autoimmunity (39). In physiologic conditions, HLA-G molecules display tissue-restricted expression. However, under certain pathological circumstances - such as viral infection, malignancy, transplantation, oxidative stress, hypoxia, inflammatory and autoimmune disorders - the expression of these molecules can be upregulated by interferons, interleukin 10, and other factors, such as microRNAs (36, 40, 41), and favour anti-inflammatory Th2-type responses, possibly in order to protect tissues against excessive inflammatory aggression (42). It is important to add that pro-inflammatory responses have also been described for HLA-G molecules, and the HLA-G homodimer has been shown to induce secretion of the proinflammatory cytokines interleukins 6 and 8, as well as tumor necrosis factor alpha from both decidual macrophages and NK cells (43).

### Classical Versus Non-Classical MHC Molecules

Classical MHC genes are highly polymorphic, particularly in the peptide-binding domains. Polymorphisms are thought to be an adaptative evolution in response to pathogen exposures, driving selection on MHC, with resultant allele frequency changes in different populations (44). While classical class I genes (*HLA-A, -B, -C*) are highly polymorphic and mainly present antigenic peptides to CD8T cells, the nonclassical major HLA class Ib genes (*HLA-E, -F, -G, -H*) exhibit lower polymorphism and limited tissue distribution, and are typically recognized by alternative immune receptors or, are only expressed under certain stimuli (45), having important immunoregulatory and nonimmunological functions, such as stimulation or inhibition of natural killer (NK) cells (46). The nonclassical MHC class I genes are considered evolutionary conserved, and in addition to interaction with CD8 and NK cells, non-classical MHC molecules also interact with receptors such as CD94/NKG2 to modulate immunity (47).

**Figure 1:**
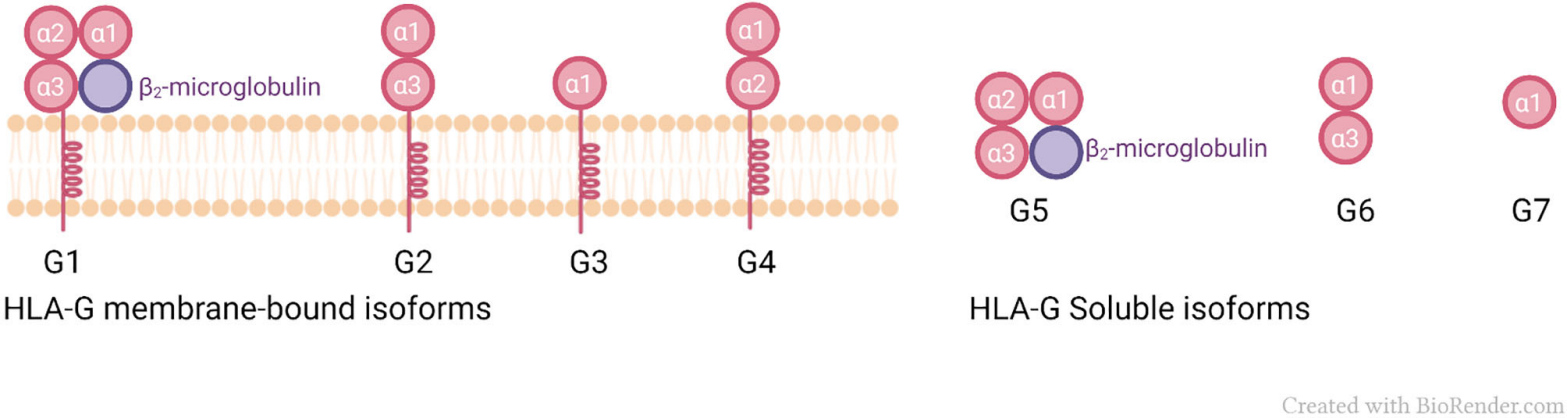
Membrane-bound and soluble isoforms of HLA-G.

HLA-G shows 86% amino acid similarity with classical class I genes, with the main difference being due to the occurrence of a stop codon in exon 6, resulting in a shorter protein, compared to classical class I molecules (48). HLA-G exhibits low polymorphism (49, 50), and a currently known polymorphic diversity of only 88 alleles, including 4 null and 2 questionable alleles (51). Seven isoforms of HLA-G produced by alternative splicing of the primary transcript (50) have been described, four of which are membrane-bound (HLA-G1, -G2, -G3, and -G4) and three of which are soluble molecules (HLA-G5, -G6, and -G7) (52). Additionally, HLA-G1 may form a soluble dimer. It was also proposed that novel HLA-G isoforms may be formed in specific conditions, such as carcinomas (36, 53). While HLA-G1 has the classical class I domains: (α1, α2, α3) and is associated with β_2_m; HLA-G2 is composed of α1 and α3 domains only, and HLA-G3 is composed of only α1. HLA-G4 has domains α1 and α2; HLA-G5 and HLA-G6 are soluble isoforms and have the same extracellular globular domains of their membrane-bound counterparts: HLA-G1 (α1, α2, α3, associated with β_2_m) and HLA-G2 (α1, α3), respectively (54), **Figure 1**.

Despite the significance of HLA-G in immunomodulation, pregnancy maintenance and graft survival, surprisingly little is known about the molecular structure and dynamic behavior of the HLA-G isoforms (54). In fact, to date, the Protein Data Bank (PDB) has only made available a few crystal structures related to HLA-G1 (54, 55). Recently, with the use of structural modeling, Arns et al. (54) have provided insight into the structure and dynamic behavior of the membrane-bound HLA-G1, as well as for the soluble HLA-G1 dimer. The authors demonstrated a “tilting” movement for membrane-bound HLA-G and have argued that the location of major leukocyte receptor binding sites in the α3 domain, close to the membrane, could restrict these interactions with ILT2 and ILT4 receptors. By contrast, full rotational freedom was demonstrated for soluble HLA-G1 dimer, which would allow a large conformational flexibility for this molecule in solution, resulting in larger exposure of the binding sites and additional binding possibilities, as compared to its membrane-bound counterpart, which could explain the observed higher affinity of soluble HLA-G1 dimer to ILT2 and ILT4 receptors as compared to the membrane-bound HLA-G1 (54). These findings illustrate the structural diversity of HLA-G molecules, with associated impact on their functional versatility.

In MHC Class I molecules, the α1 and α2 domains fold into an oval-shaped groove that can accommodate short peptides (usually 8-10 amino acid residues). Class II molecules are formed jointly by two different gene products, α and β chain and have a peptide binding groove surrounded by two parallel helical structures (α1 and β1), which can accommodate larger peptides (commonly 13-25 amino acid residues) (28).

**Figure 2:**
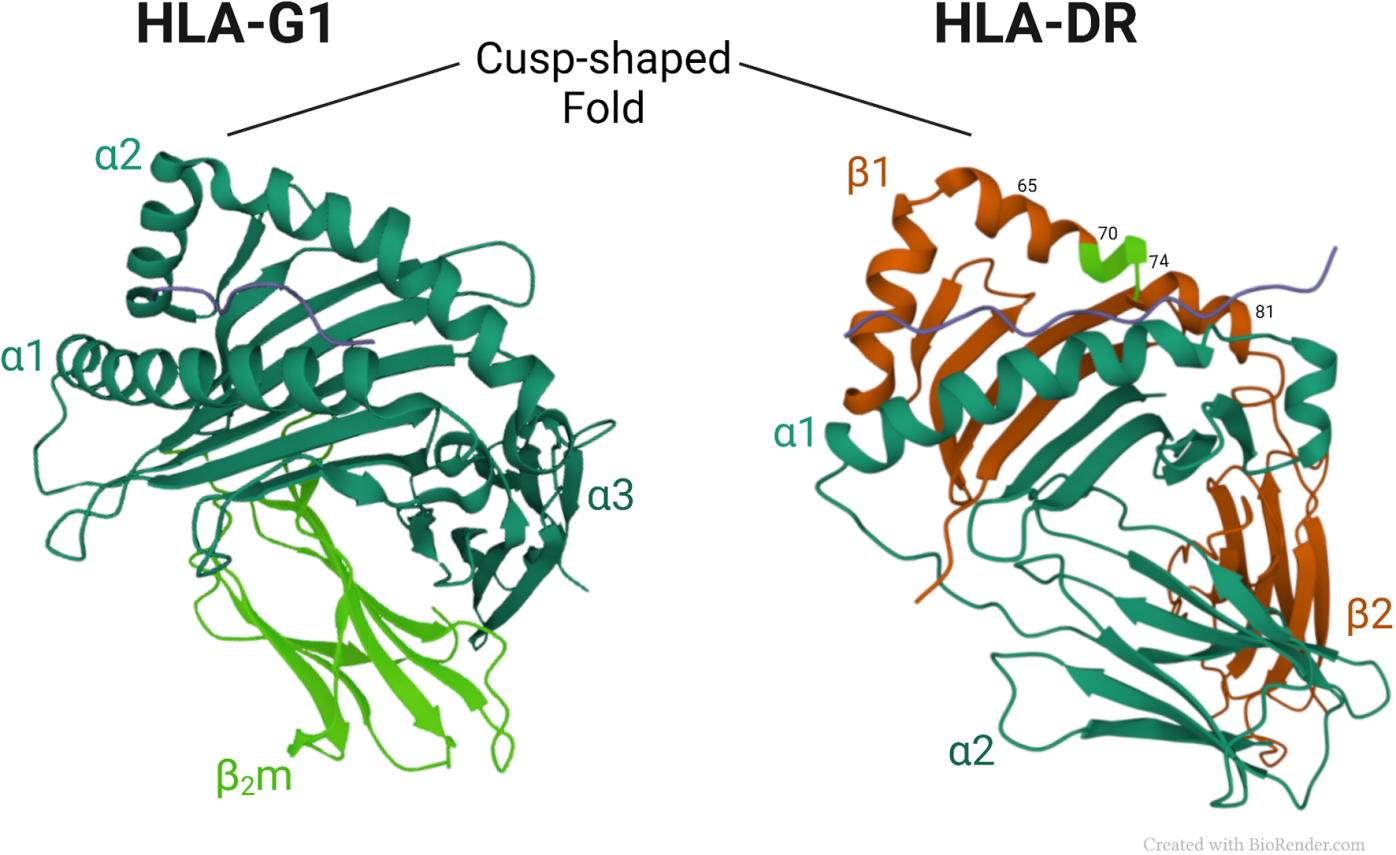
Crystal structures of HLA*-G1* (pdb, 3KYN) and *HLA-DR* (pdb, 1A6A) (56), focusing on their respective cusp folds. Amino-acids at positions 70–74 of the DRβ-chain are highlighted in light green.

**Figure 2** shows a comparison of the three-dimensional structures of HLA-G1 and HLA-DR molecules. Of note, is a similar cusp-shaped fold in the α2 domain of HLA-G1, and the β1 domain of the HLA-DR molecule, despite their markedly different chain composition, and considerable evolutionary distance. This suggests that the respective conformationally homologous regions may have conserved their common shape through evolution to retain a fundamental functional advantage that this region provides. Below we discuss the possibility the cusp region has retained its shape through evolution to preserve important ligand functions.

The MHC system is not without flaws, and HLA-associated diseases have been described since the 1960s. Over the years it has been realized that the HLA region in the human genome has the greatest known number of associated diseases, such as type 1 diabetes (57), RA (58), Alzheimer’s disease (59), schizophrenia (60), and many others (2). Recently, a study evaluated HLA type-I genotypes with outcomes in course of COVID-19, and demonstrated a high risk for death of adults with the presence of the HLA-A*01:01 allele (61), highlighting the importance of the HLA phenotype also in acute illnesses. Although genes outside of the MHC locus also encode antigen-presenting molecules (62), this review will focus on HLA-related molecules.

## HLA-Disease Association - Canonical Hypotheses

The HLA complex is one of the most extensive and polymorphic gene groups in the human genome. It has undergone extensive refinements through evolution to assure effective protection against foreign organisms on the one hand, while preserving the health of the host, on the other. Paradoxically, however, it is the very same group of genes that is implicated as risk factors in a long list of diseases that affect every organ system and tissue in the body. How has it happened that a safeguard that has been so meticulously perfected to police and defend the body against disease turned into a culprit? This enigma, which has been at the focus of scientific debate over the last 5 decades, has generated multiple different hypotheses that offer a variety of answers to this perplexing question. Given that the best-known function of HLA molecules is to present peptide antigens to T cells, the “canonical” hypotheses are focused on various AP flaws.

Among the most widely discussed canonical hypotheses are reactivity to self-antigens due to molecular mimicry with foreign antigens; skewed T cell repertoire selection; reactivity to altered self-antigens; and association through LD. Additional theories concerning the mechanistic basis of HLA-disease association are discussed in a recent review article (63).

### Molecular Mimicry With Foreign Antigens

**Figure 3:**
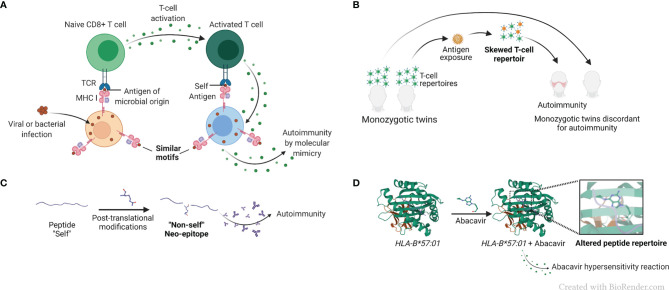
AP-based hypotheses for the mechanistic basis of HLA-disease association. **(A)** Simplified representation of the molecular mimicry hypothesis. Molecular mimicry occurs when the similarities between foreign peptides and self-peptides are such that auto-reactive T cells are activated, triggering autoimmunity. **(B)** Simplified representation of the skewed T cell repertoire hypothesis in monozygotic twins. Naïve T cells are activated after recognition of non-self-antigens presented by MHC molecules, and T cell repertoire is skewed towards new antigen-driven specificities. **(C)** Representation of the reactivity to altered self-antigens caused by protein citrullination. In the reactivity to altered self-antigens hypothesis, proteins that were previously tolerated by the immune system suffer changes and become neo-epitopes and recognized as “non-self”, breaking immunological tolerance. **(D)** Representation of a drug-associated HLA modification caused by abacavir, pdb, 3VRJ (64).

The term “molecular mimicry”, also known as “immune cross-reactivity”, refers to mistaken identification by the immune system of a self-antigen as foreign, due to their structural resemblance, as schematically represented in **Figure 3A**. The concept, invoked as far back as 60 years ago (65),was rationalized based on primary sequence homology between human and microbial antigens (66), as well as cases of test tube cross-reactivity of antiviral monoclonal antibodies with host tissues and cells (3, 67). Molecular mimicry has been implicated in many diseases and autoimmune disorders, such as COVID-19 (68), uveitis (69) and multiple sclerosis (70), among other conditions. A recent study has implicated molecular mimicry as an underlying mechanism of protection against RA by certain *HLA-DRB1* alleles encoding for a DERAA sequence in the cusp region due to a tri-lateral sequence homology among a self-protein named vinculin, microbial proteins, and the products of the protective *DRB1* alleles (71). However, the premise of this complex hypothesis, which postulates antigenic mimicry among proteins derived from three different sources (microbial, vinculin and HLA-DR), as well as AP by an HLA-DQ that is in LD with the protective *HLA-DRB1* allele has raised some questions (23, 72).

Importantly, despite much effort in the past decades, a direct link between molecular mimicry and autoimmunity remains elusive, and the identity of putative antigens have not been convincingly and reproducibly demonstrated in HLA-associated diseases (8). It is noteworthy that a series of studies with viral and bacterial proteomes demonstrated extensive overlaps between human and microbial motifs, suggesting that this homology is such that it should have led to a frequency of autoimmunity much greater than the 5% currently reported (73). This analysis, and lack of direct cause-effect data to implicate molecular mimicry in human autoimmune diseases strongly suggest that molecular mimicry does exist; however, it is unlikely to be a significant pathogenic factor in autoimmunity (74–77).

### Skewed T Cell Repertoire Selection

T cell repertoire is shaped through a series of positive and negative thymic selection processes, leading to deletion of self-reactive T cell receptors (TCR) in the context of self-MHC (4), (**Figure 3B**). Studying monozygotic twins, Utz et al. demonstrated distinct T cell repertoires in multiple sclerosis-discordant twin pairs (78). The skewing was observed in peripheral blood lymphocytes stimulated both with a putative target self-antigen, myelin basic protein, as well as with an unrelated foreign antigen tetanus toxoid, demonstrating an antigen-nonspecific variations in T cell repertoires between the discordant twins (78). These findings were interpreted to suggest that factors other than genetics exert influence on an individual’s T cell repertoire in multiple sclerosis, an HLA-associated disease. Additionally, one significant limitation of the T cell repertoire selection hypothesis is that despite much effort, clonality of antigen-specific T cells is yet to be convincingly demonstrated in HLA-associated diseases.

### Reactivity to Altered Self-Antigens

Alterations of self-antigens might render them recognizable as foreign by the immune system. A classic example of a post-translational modification is protein citrullination (5), which has been proposed as a triggering event in RA disease development (79). Protein citrullination is an important process necessary to maintain physiological function of several proteins. In patients carrying *HLA-DRB1* alleles encoding for the shared epitope (SE) (80), anti-citrullinated protein antibodies (ACPA) are useful diagnostic and prognostic marker (81). It has been hypothesized that exposure to environmental triggers, such as cigarette smoke (82) leads to accelerated protein citrullination, which in turn become a target for auto-antibodies (5, 83), (**Figure 3C**). The increased risk of RA development in smokers has been established for years (84), especially amongst individuals carrying particular *HLA-DRB1* alleles encoding the SE (85, 86). Although it has been demonstrated that some citrullinated proteins have a higher binding affinity to the SE than their native counterparts (87), that observation is not universal and does not necessarily govern T cell response (88). Thus, it has been argued that presentation of citrullinated peptides is unlikely to be the sole link between the SE and generation of ACPA (88, 89). It has also been hypothesized that cigarette smoke facilitates protein citrullination in the lungs of susceptible individuals, resulting in generation of autoantibodies directed against these citrullinated proteins (90, 91). However, this hypothesis does not explain the increased risk of RA development in children of women who smoked during pregnancy (92).

### Association Through Linkage Disequilibrium

The HLA complex is characterized by extensive LD, meaning a non-random (either higher or lower) association of alleles at different loci (93). LD frequently renders the identification of the causative genes in disease associations more challenging (94). It should be mentioned, however, that associations through LD do not provide a mechanism, they just identify spurious associations while the mechanistic basis remains unknown. Indeed, erroneous HLA-disease associations have been established due to LD, such as in the case of narcolepsy, where predisposing factors had been mistakenly attributed to HLA-DRB1*15, and later were found to be associated to the *HLA-DQ* genes that are in LD with DRB1*15 (6). These inaccurate associations are becoming less common, as genetic and epidemiological research technologies have evolved and are being steadily optimized (95).

### Drug-Induced HLA Modifications

Certain small-molecule drugs have been shown to bind to specific HLA proteins and cause modifications that result in altered binding repertoires and subsequent HLA-associated adverse drug reactions. A case in point is the abacavir hypersensitivity syndrome, developed by individuals carrying the HLA-B*57:01 allele. It has been demonstrated that the drug used in HIV therapy, binds non-covalently to the peptide-binding cleft of HLA-B*57:01-coded protein, resulting in a modification of its peptide-binding repertoire, which may lead to presenting endogenous peptides (96), (**Figure 3D**). The knowledge of this HLA-associated hypersensitivity has prompted a change in HIV treatment guidelines (97), with resultant marked decline in hypersensitivity occurrence (98).

## HLA-Disease Association - Noncanonical Hypotheses

While most hypotheses concerning HLA-disease association are centred on AP, evidence in support of AP-independent mechanisms has been presented as well. For example, certain HLA-B*27 alleles are found in more than 90% of patients with ankylosing spondylitis and explain 20.1% of the heritability of the disease (99). It has been found that amino acid Cys67 in such HLA-B27 molecules favors an open conformation of the molecule, which is a stronger ligand for leukocyte Ig-like receptor B2 (LILRB2) than other HLA class I molecules (100). It has also been demonstrated that HLA-B27 molecules are predisposed to misfolding (101, 102), generating disulfide-linked homodimers, which are recognized by KIR 3DL2 on CD4^+^ T_H_17 cells, and stimulate the production of IL-17 (103). Notably, misfolded proteins have been shown to accumulate in the endoplasmic reticulum and activate the unfolded protein response, an aberration which has been implicated in the pathogenesis of rheumatic diseases (102).

Another example of non-canonical hypothesis is the modulation of cell surface receptors by MHC molecules. An example is in the case of the human cytomegalovirus (HCMV) infection, which has been demonstrated to modulate HLA-C, favoring a potent KIR2DS1-mediated NK cell activation (104).

### The MHC Cusp Theory

In addition to the individual limitations discussed above, AP as a sole causative factor in HLA-associated diseases is difficult to reconcile with several fundamental epidemiologic and pathogenic characteristics of those diseases:

1. Many of the diseases that are known to associate with specific HLA alleles display neither autoimmune basis, nor evidence of AP. For example, HLA allele DQB1*06:02 is found in almost all patients with narcolepsy type 1 (105), yet convincing, cause-effect relationships between the DQB1*06:02 allele and autoantibodies or AP have not been shown in this disease, which is caused by an abnormal neurotransmission.
2. A single allele can associate with multiple diseases with distinct pathogeneses or target tissues. For example, HLA-DRB1*03:01, has been associated with systemic lupus erythematosus (106), Graves’ disease (107), and type 1 diabetes (108), to name just few. Likewise, the aforementioned narcolepsy-associated DQB1*06:02 allele is also a genetic risk factor for an unrelated disease, multiple sclerosis (109).
3. Particular alleles can offer disease-susceptibility across species, for example, the SE sequence has been found to associate with human RA (110), as well as canine arthritis (111) and murine models of the disease (112). It is hard to conceptualize the evolutionary advantage of such trans-species AP-based susceptibility factor among species that have vastly different adaptive immune system antigen recognition repertoires.
4. The impact of HLA allele-dose on disease severity by specific alleles does not fit well with mainstream AP-based concepts. For example, it has been demonstrated that in RA, allele-dose effects can determine disease incidence, severity, age of onset and concordance rates in RA-discordant monozygotic twins (113, 114). The effect of homozygosity is incongruent with AP-based clonal expansion. Moreover, HLA-DR heterozygosity has actually been shown to increase disease susceptibility in several conditions, including systemic lupus erythematosus (115), and type 1 diabetes (116).

We put forward the MHC Cusp theory a decade ago (9) to provide an alternative explanation to the aforementioned inconsistencies that cannot be convincingly answered by the AP-based hypotheses. The theory postulates that: *“HLA molecules encode ligands in one of their hypervariable regions, designated a “cusp” based on its three-dimensional cusp-like conformation. Under certain environmental and background gene conditions, these cusp-ligands can interact with non-major histocompatibility complex (MHC) receptors thereby activating aberrant cell signaling events that cause disease development”* (9).

To empirically examine the MHC Cusp theory, our group has so far focused on two representative *HLA-DRB1* alleles known for their association with autoimmune diseases. The SE, a five amino acid motif (QKRAA, QRRAA or RRRAA) in the cusp 70-74 region of the DRβ chain coded by RA-associated *DRB1* alleles, and is strongly associated with RA susceptibility (110), was the first to be studied. Our findings to date have demonstrated that the SE acts as a signal transduction ligand that interacts with cell surface calreticulin (CRT) and activates signaling events, which facilitate pro-arthritogenic and bone erosive changes (10–23). We have demonstrated that human lymphoblastoid B cell lines carrying SE-coding HLA-DR alleles triggered spontaneous production of nitric oxide (NO) (10). We later demonstrated that the SE caused increased NO and reactive oxygen species (ROS) production, and facilitated osteoclast differentiation in RAW 264.7 cells, bone marrow cells (BMCs) derived from DBA/1 mice, and in healthy human peripheral blood mononuclear cells (PBMCs). The SE was also found to promote increased production of interleukin (IL)-6 and tumor necrosis factor alpha (TNF-α), and enhanced the differentiation of receptor activator of NF-kB ligand (RANKL)-expressing IL-17 producing T cells, causing Th17 cell-mediated pro-osteoclastogenic effects independent of antigen presentation (15).

Our studies with this cusp ligand have also provided a mechanistic basis for the long-observed association between RA, cigarette smoking and carriage of SE-coding alleles (117), by demonstrating that in the presence of aryl hydrocarbon receptor (AhR) agonists - environmental pollutants found in cigarette smoke - the activation potency of the SE-mediated pathway is amplified synergistically with resultant augmented inflammatory response and bone erosive damage that lead to more severe experimental arthritis in mice (21). In a subsequent study, we demonstrated in RAW 264.7 cells, that lipopolysaccharide (LPS) facilitated cell surface translocation of CRT, which in turn enabled increased SE-activated calcium signaling and activation of peptidylarginine deiminase, increasing cellular abundance of citrullinated proteins. Intraperitoneal administration of LPS in transgenic mice carrying a human SE-coding *HLA-DRB1* allele caused increased serum levels of TNF-α and ACPA production, as well as terminal phalanx bone destruction (22), thereby suggesting a mechanistic basis for the long documented association between RA and protein citrullination. Additionally, we have demonstrated that the interaction between SE and its receptor, CRT, is facilitated by citrullination of the latter (16). Thus, the Cusp theory provides a multifaceted, AP-independent model of HLA-disease association and its interaction with environmental factors.

The second cusp motif that we have studied was the 70-DERAA-74 sequence, which is coded by *DRB1* alleles known to protect against RA and several other autoimmune conditions (118–122). An AP-based hypothesis to explain the contrasting effects of the SE and PE has been offered based on the charge of critical amino acid residues coded by these two functionally distinct alleles. It has been proposed that the positively charged residues lysine and arginine in the SE would favor the binding of citrullinated proteins, while the PE, which contain negatively charged residues, can bind to both arginine and citrulline residues (123). However, the observation that other HLA molecules (e.g. DQ), and peptide binding pockets outside of the 70-74 region in HLA-DR molecules could accommodate citrullinated peptides as well (123), suggests that the interactions between HLA molecules and citrullinated peptides associations are more complex.

Given the limitations of the canonical, AP-based hypotheses to provide convincing explanation for the effects of protective *DRB1* alleles, we have taken a cusp-based experimental approach. As discussed above, an amino acid sequence motif 70-DERAA-74 in the DRβ chain shows a protective effect (118, 123). Using short synthetic peptides corresponding to this cusp region, we have demonstrated that these AP-incompetent peptides activated transcriptomes that are consistent with anti-inflammatory (M2) macrophage polarization. A diametrically opposite effect was observed with synthetic peptides corresponding to the SE motif, which activated a pro-inflammatory macrophage (M1) transcriptome (23).

**Figure 4:**
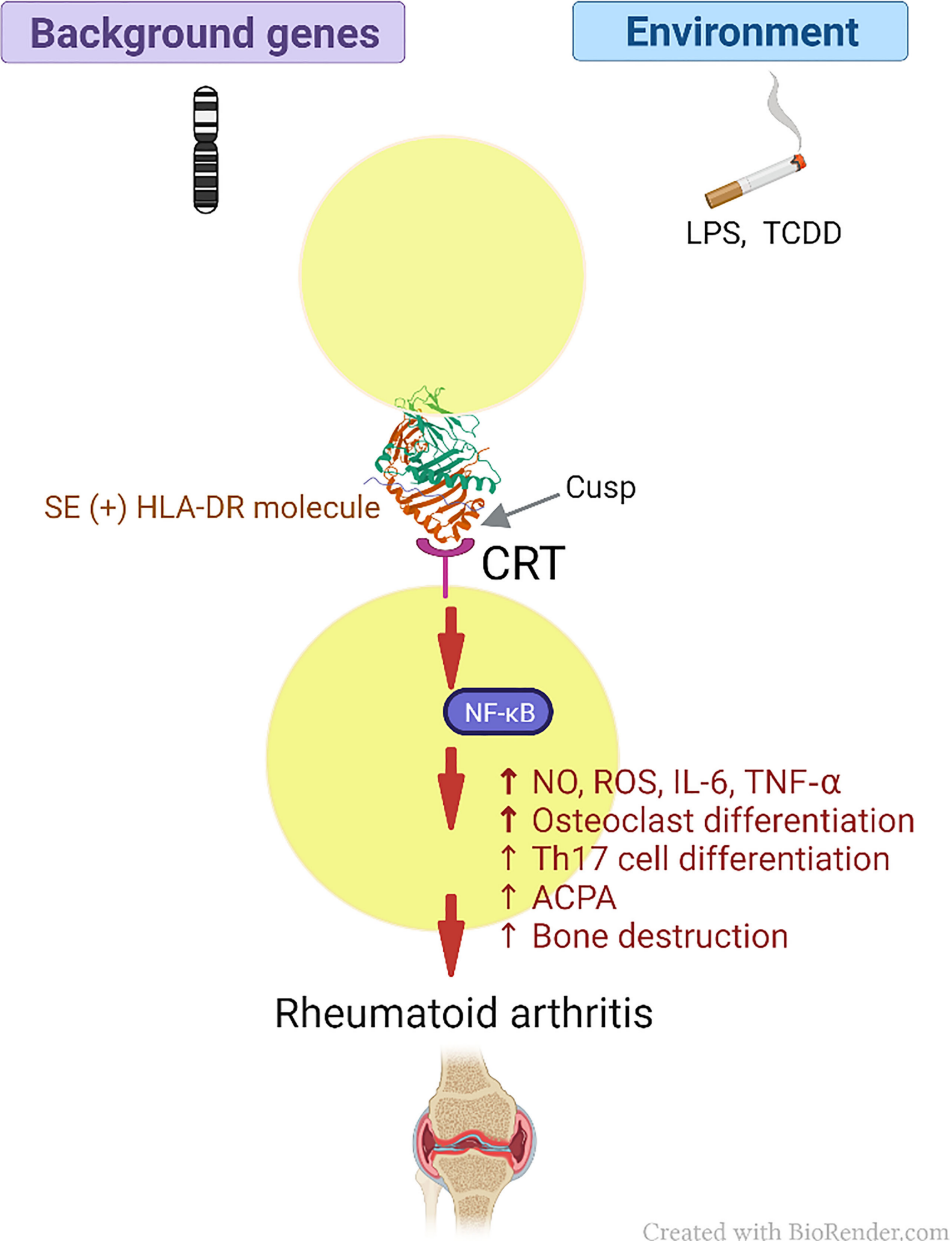
Schematic model of the MHC Cusp theory (as exemplified by the effect of the SE in RA). The cusp region of RA risk-conferring HLA-DR molecules contains an allele-specific physiologic signal transduction ligand (SE) that can help polarize the immune response through interaction with CRT, which acts as a cell surface receptor, with resultant activation of immune modulating pathways. Depending on permissive background genes and environmental influences such as pollutants, the otherwise physiologic pathways can activate cellular events that lead to the onset of RA (please see text for details). CRT, calreticulin; LPS, lipopolysaccharide; TCDD, 2,3,7,8 -Tetrachlorodibenzo-p-dioxin; SE, shared epitope; NF-κB, Nuclear factor-κB; RA, rheumatoid arthritis; NO, nitric oxide; ROS, reactive oxygen species; IL-6, interleukin 6; TNF-α, tumor necrosis factor alpha; Th17, T helper 17; ACPA, anticitrullinated cyclic peptide antibodies.

Thus, our findings provide empirical evidence in support of an AP-independent mechanism that contributes to HLA-disease association. It is important to clarify that the MHC Cusp theory does not aim to supplanting the role of AP, it rather demonstrates that in addition to their well-studied classical AP functions, MHC molecules express cusp-region epitopes that under certain environmental conditions can trigger activation of disease-facilitating pathways. In summary, the amino acid sequence in the cusp-region of these molecules would govern the binding specificity of these binding sites, while the three-dimensional cusp-like structure makes it more accessible for interaction with cognate receptors. A proposed scheme of the role of MHC cusp ligands in disease pathogenesis is shown in **Figure 4**.

### A Summary of the Experimental Evidence Supporting the Ligand Function of the SE

Our research to date, using a range of approaches and techniques has provided considerable evidence in support of the SE effects as a cusp ligand. Our early findings involved lymphoblastoid B cell lines carrying SE-positive or -negative *HLA-DRB1* alleles, as well as L cell transfectants that carry allele-distinct HLA-DR molecules, or fibroblast cell transfectants expressing HLA-DR molecules with single amino acid point mutations. In all cases, SE-positive cells showed increased NO production. Moreover, the signaling effects were found to be independent of any cellular context, since cell-free tetrameric HLA-DR molecules that possess a SE-positive DRβ molecules stimulated fibroblast cells to produce higher levels of NO compared with cells stimulated with a control HLA-DR tetramer. Likewise, multimeric hepatitis B core proteins engineered to express the SE cusp region, but not the same region encoded by a SE-negative allele, stimulated NO production in fibroblasts. Furthermore, cell-free soluble synthetic peptides corresponding to the cusp region of SE-positive HLA-DR molecules triggered increased NO levels in class II HLA-negative cells (10).

Consistent with the known association between the pro-oxidative effect of NO and the proposed role of oxidative stress in the pathogenesis of RA, we have demonstrated that cells exposed to cell-surface-expressed, as well as cell-free soluble SE-positive cusp regions showed increased ROS levels, and higher vulnerability to oxidative DNA damage in an NO-dependent fashion. Using single amino acid substitutions, we have identified a sequence motif of Q/R-K/R-X-X-A that is critical for the SE cusp region signaling effect (11).

In other studies, we identified allele-specific cusp ligand-triggered activation of cellular calcium increase, and activation of the NF-kB pathway (10, 11, 21, 22). We have also demonstrated synergistic cross talk between the SE and the AhR pathways (21) and allele-specific distinct cytokine and chemokine production profiles by different cusp region ligands (14, 15, 17, 23). Additionally, cell activation by cusp region ligands was found to trigger allele-specific signature transcriptomes (23).

Allele-specific signal transduction pathway modulation by the SE versus the PE has been mapped, demonstrating that under M1-polarizing conditions, bone marrow-derived macrophages (BMDM) of SE-positive transgenic mice demonstrate a cascade of signaling events, involving pSHIP1 activation, inhibition of Pi3k and pAkt, and NF-κB activation, leading to enhanced M1 polarization. In contrast, in BMDM of PE-positive transgenic mice, a mirror image of signaling event is found, leading to decreased M1 polarization (23). Under M2-polarizing conditions, BMDM of PE-positive transgenic mice show a tandem upstream pathway effect through activation of pAkt and pStat6, which activate transcription of M2-polarization genes *Arg1* and *Yam1*, respectively, with resultant enhanced M2 polarization. In contrast, in SE-positive transgenic mice, effective M2 polarization is inhibited by sustained NF-κB activity (23).

Relevant to the role of SE in RA disease pathogenesis, we demonstrated allele-specific RA-associated cytokine and chemokine production profiles by the SE, as compared to other allelic cusp ligands (14, 15, 17, 23). In addition, the SE ligand was found to specifically facilitate Th17 and inhibit Treg differentiation (14). It also polarized macrophage differentiation toward a pro-inflammatory subset (M1), with a diametrically opposite effect by the PE cusp ligand, which was found to facilitate differentiation of anti-inflammatory subset of macrophages (M2) (23). Another RA-relevant finding is the pro-arthritogenic effect of synthetic peptide and peptidomimetic SE ligands *in vivo* and potent activation of osteoclast differentiation and bone erosive effects, both *in vitro* and *in vivo* (15, 18).

To identify the SE signal-transducing cell surface receptor, we used a combination of affinity chromatography, cell-binding assays, surface plasmon resonance, and time-resolved fluorescence resonance energy transfer techniques to demonstrate specific interactions between the SE ligand and CRT at a cell-free level. The interaction was confirmed at the cellular level by studying CRT-KO, as well as CD91-negative cells, along with antibody-mediated blocking experiments and reconstitution techniques. Thus, these experiments demonstrated that SE-triggered cell signaling effects depend on CRT and its known co-receptor, CD91 (12).

We have mapped the SE binding site on CRT to a discrete region on CRT P-domain. This was accomplished by using surface plasmon resonance-based experiments with domain deletion mutants, and a photoactive cross-linking method. Based on in silico-based docking interactions between the SE in its natural conformation and the CRT P-domain, followed by site-directed mutagenesis we identified the amino acid residues in the binding site that are critical for binding to the SE ligand and effectively transducing signaling (13).

Based on the molecular details of SE interaction at the specific binding site on CRT, we designed synthetic SE agonists that mimic the SE effects, as well as antagonists that block SE-CRT interaction. Based on those data, we have successfully designed different synthetic molecules that specifically target the interaction site and facilitate arthritis or inhibit it in an experimental model of arthritis (15, 18, 112, 124).

Thus, we have deciphered key molecular mechanisms associated with the cusp ligand SE effects *in vitro* and *in vivo* and have embarked on reducing this knowledge to practice.

## Parallels Between HLA-G and the MHC Cusp Theory

HLA-G and the MHC Cusp theory demonstrate functional and conceptual parallels. Reminiscent of the Cusp theory that has identified several *HLA-DRB1* alleles which code for ligands that activate signaling events that result in transcriptional and functional immune modulation, HLA-G activity has been shown to involve ligands that exert profound immune modulatory effects. Like the Cusp theory, it has been proposed that in at least some HLA-G isoforms, AP is not required for their functional activities (125), and both Cusp epitopes and certain HLA-G molecules involve activation of specific signaling events. The following are four areas illustrating salient functional and structural characteristics, that illustrate the parallels between the MHC Cusp theory and HLA-G.

### Immune Modulatory and Autoimmune Disease Effects

As discussed above, the MHC Cusp theory offers a non-AP mechanistic basis for HLA-associated diseases and health traits. The cusp region in class II HLA molecules encompasses the third allelic hypervariable region of their β chain, with which numerous epidemiologic studies have identified allele-specific associations in many autoimmune diseases, such as RA, systemic lupus erythematosus, type 1 diabetes, and many other conditions. While AP might explain some of the associations, it is important to note that in most HLA-associated diseases, the identities of the self or foreign antigens and conclusive evidence to substantiate AP-based mechanism is limited despite decades of research effort. Based partly on this reality, as well as the promiscuous associations of many alleles with diseases and conditions that have no common pathogenetic, mechanistic, or tissue distributions, we have previously proposed that notwithstanding a putative role of AP, the Cusp theory may provide a mechanistic framework for at least some allele-specific disease association. Using two emblematic *HLA-DRB1* alleles known to associate with autoimmune disease susceptibility or protection, DRB1*04:01; DRB1:04:02, we have shown that AP-incompetent ligands - peptide sequences unable to perform antigen presentation - corresponding to the cusp regions of these alleles trigger disease-relevant transcriptional, signaling, cell activation and disease phenomes (14, 15, 19, 21–23).

Like class II HLA alleles, HLA-G products have been shown to associate with diverse and mechanistically unrelated autoimmune and neoplastic diseases, and to play a crucial role in preserving fetal-maternal tolerance, independent of AP. The best characterized roles of HLA-G are in implantation and maintenance of pregnancy (126, 127), and tolerance to solid organ allografts (35). In the context of the Cusp theory, however, it is important to mention that HLA-G immune modulatory effect has been documented in several autoimmune conditions as well. For example, polymorphisms in the HLA-G gene have been found to be a significant risk factor for type 1 diabetes (128), systemic lupus erythematosus (129, 130), RA (131), experimental autoimmune uveitis (132), systemic sclerosis (130), as well as multiple sclerosis and several autoimmune skin conditions (133), asthma (134) and Graves’ disease (135), however, it is important to mention that results for associations between HLA-G and diseases are often times contradictory or inconclusive, and warrant further investigation.

The promiscuous immune modulatory effects of HLA-G in multiple diseases that lack common tissue distribution or pathogenesis suggest a fundamental, antigen-nonspecific effects, as discussed below.

### Ligand Effect Versus AP

Like classical class I HLA molecules, certain HLA-G isoforms are folded in a three-domain conformation, associate with β_2_m, possess a peptide-binding groove, and interacts with CD8+ T cells (136). Further, HLA-G has been reported to carry natural peptides with characteristic groove-binding motifs (137), and has been implicated in HLA-restricted antigen-specific cytolytic CD8+ repertoire selection (138).

However, different from class Ia molecules, HLA-G has a limited tissue distribution and a short intracytoplasmic domain, it shows a low level of polymorphism, and its mRNA is spliced into multiple isoforms. Further, HLA-G cleft peptides are positioned in a constrained mode of binding, which is distinct from the mode that characterizes peptide groove binding in class Ia HLA molecules (139).

Based on these considerations and others, it has been proposed as far back as the mid-1990’s that AP might not be the only - or even the primary - function of HLA-G molecules (125, 140). As discussed above, various HLA-G isoforms have been shown to interact with immune receptors such as KIR and activate in trans several functional effects, including modulation of innate and adaptive immunity, anti-viral, anti-tumor, as well as non-immune functions. Those trans-activation events involve defined signaling pathways, which are detailed elsewhere (127).

Potentially relevant to HLA-G-activated pathways, the PE, coded by *HLA-DRB1* alleles known to associate with protection against several autoimmune diseases (118, 141), can activate signaling and transcriptional events that characterize immune tolerance (23). Although the identity of the PE receptor is not yet known, our findings indicate that the PE ligand activates S473Akt phosphorylation with downstream effects on the NF-κB pathway, and M2 macrophage polarization, reminiscent of signaling events observed with HLA-G (142, 143). Thus HLA-DR cusp region ligands in the form of short linear synthetic peptides, which lack AP capacity, can activate cascades of signaling and cell differentiation events with potential effects on health and disease processes.

### Topologies of Receptor-Ligand Interactions

The MHC Cusp theory has been prompted by growing evidence linking a specific region on the MHC molecule, which despite substantial polymorphism and evolutionary distance, is conformationally conserved in the entire MHC family of molecules and other MHC fold proteins (144). As previously discussed (8, 9), that region, which was named by us “cusp” due to its conformationally conserved cusp-like structure, appears to be a hub of epitopes that interact with various non-MHC receptors and activate functional effects. Those receptor binding epitopes have been identified across the MHC family regardless of the antigen presentation capabilities of the parent molecules, as follows.

In class II HLA molecules, the cusp region which shows immune modulating ligand properties has been mapped to the amino acid residues 65-79 in the β1 domain, which forms an α helix with an ‘upward kink’ (**Figure 2**). The conformationally analogous region to the MHC class II cusp region in class I molecules is in the α2 domain, where a similar ‘kink’ is found. That region in Class I is enriched in ligands as well. For example, in class Ia molecules, such as HLA-A (145) and HLA-C (146) the cusp region has been shown to bind natural killer cell inhibitory receptors, such as KIRDL1, in a tandem binding geometry, where the KIR domain D1 binds to the α1 domain of the HLA molecule, while domain D2 interacts with the α2 domain, at the cusp region. Equivalent cusp-region binding topologies were found between HLA-C molecules and KIRDL2 and KIRDL3 (147). Cusp-region interaction involvement has been found in the class Ib molecules as well. Reminiscent to the above-mentioned binding topology of class Ia HLA molecules, a tandem of CD94-NKG2A binding sites has been mapped, respectively, to the α1 and α2 domains of the class Ib HLA-E molecule (148–150). Noteworthy, cusp region binding sites have been found also in non-antigen presenting members of the extended MHC family, such as the hereditary haemochromatosis protein HFE, which interacts in its cusp region with transferrin receptors (151). Another example is mouse M10, a pheromone receptor-associated MHC molecule with an open and empty groove whose cusp region interacts with odorant receptors of the vomeronasal organ (152).

Given the common structural and functional properties between HLA-G and other HLA molecules (144), and since evidence exists for an immune receptor-binding site on the α2 domain of the latter, it is reasonable to predict that the HLA-G could contain an equivalent receptor binding site in the cusp domain as well. Evidence to support this scenario, however, is inconclusive. On the one hand, specific polymorphic residues in the HLA-G1 α2 domain were found to impact HLA-G NK cell recognition and immune regulation (153), however, a recent study has concluded, based on co-crystallization experiments, that there was no evidence to support a role for interactions between immune receptors and the HLA-G1 α2 domain (154). It should be added that confirmatory structural analyses, or functional studies are yet to be published, particularly considering the interplay between soluble and membrane-bound isoforms. Given the exquisite sensitivity of crystallography studies to even minor physicochemical alterations, it is still possible that the final word on whether the HLA-G α2 cusp region plays a role in ligand-receptor immune modulating interactions is yet to be said.

### Therapeutic Implications

One of the practical outcomes provided by the MHC Cusp theory is the prospect of identification of specific ligand-receptor targets for therapeutic intervention. A practical example of the therapeutic potentials was realized upon the identification of the SE binding site on CRT by our group (13). That finding has opened the door to the development of an antagonistic ligand, which showed potent therapeutic effect in mice with experimental erosive arthritis (112). Likewise, the identification of the anti-inflammatory pathways activated by the PE might offer the mechanistic insights for the development of PE-mimicking ligands with anti-inflammatory properties thereby offering therapeutic protection against HLA-associated diseases, such as RA.

A similar rationale may be used for the development of HLA-G related peptides, which might hold therapeutic promise in a number of immune-mediated diseases (133) and transplantation. Additionally, a recent work has evaluated HLA-G expression in biopsy renal tissue of patients with lupus nephritis (155). That study demonstrated that the percentage of patients who did not respond to treatment was significantly lower in the HLA-G expressing group compared to the group that did not express HLA-G, suggesting a possible role of HLA-G molecules in response to treatment (155). Synthetic, HLA-G-derived molecules have already been developed for potential use to improve tolerance to organ transplants (156).

On the other hand, HLA-G expression has been shown to play a role in escape of cancerous tumours (157) and virally infected cells from immune control (52), suggesting deleterious actions of HLA-G (38), which should be carefully considered when developing HLA-G synthetic molecules as a potential therapeutic agents. Because of the involvement of HLA-G in immune evasion and consequently tumour growth, this molecule has been considered to exert an immune checkpoint function in cancer (48). Although monoclonal antibodies have shown therapeutic success in blocking other checkpoint molecules, the structural and functional diversity of the various HLA-G isoforms have posed limitations to the development of specific antibodies for immunotherapy in cancer, and although HLA-G antibodies have been developed, they commonly do not interact with all HLA-G isoforms (56). Using specific HLA-G based ligands would surpass antibody related limitations. Thus, efforts to improve understanding of HLA-G specific receptor interactions, signaling pathways and potential targets may open the door to developing inhibitory ligands for the use in cancer therapy, which holds a great potential for tumor-specificity, since HLA-G has restricted expression in healthy tissues (48).

### A Clarification

All theories, including the MHC Cusp require independent corroboration. To date, we have presented published evidence that supports AP-independent allele-specific immune dysregulation by cusp-region sequences coded by two *HLA-DRB1* alleles*: 04:01* and *04:02*. Although our Cusp theory-based work has been very well cited, other groups have not yet publicly validated the theory. For contextual purposes, it is important to note that although the MHC Cusp theory does not supplant AP-based hypotheses of HLA-disease association, it is nevertheless often incorrectly interpreted as challenging the more prevalent, AP theory. As we clarified in previous publications, and reiterated it here as well, based on literature evidence, and our own experimental data, we propose that as plausible as they are, AP-based mechanisms alone cannot explain many of the epidemiologic and immunogenetic findings in HLA-associated diseases. We submit that the MHC Cusp theory could effectively answer several of these inconsistencies. We also wish to point out that while 10 years have passed since we first invoked the MHC Cusp theory, such time delay is not uncommon. Many other unorthodox theories awaited even a longer time before they were accepted by the mainstream scientific community.

## Concluding Remarks

Decades of research into the structure-function characteristics of HLA-G, and paralleled research effort to decipher the mechanistic basis of HLA-disease association have led to better appreciation of the versatility and functional plasticity of this family of molecules. The MHC Cusp theory and HLA-G structure-function characteristics - seemingly unrelated entities - appear to display multiple parallels. Obviously, many questions are yet to be answered; however, research data gathered over the years has already uncovered insights about possible evolutionary inter-relatedness and specialization within the vast MHC group of genes. The lessons that have been learned thus far, and those that are awaiting discovery, could help to better understand the evolution and structure-function aspects of the MHC, which in turn might generate new ideas how to harness this knowledge to promote health, and cure diseases.

## Author Contributions

BMS searched the literature and wrote the manuscript. VD participated in editing the manuscript. VD also critically reviewed the article. JH conceptualized the Cusp theory and the parallels with HLA-G and wrote sections of the manuscript. All authors read and approved the final manuscript.

## Funding

Research work by the JH group that has laid the experimental foundation of the MHC Cusp theory has been supported by the National Institute of Health, USA (Grants R01AR059085, R61AR073014, R33AR073014, R01AR074930, R21DE023845, R21ES024428, R01GM088560, R21AR056786, UL1RR02498, R21AR55170, R01AR46468 and Contract HHSN273201600123P), as well as by Rheumatology Research Foundation Innovative Research Grants, and Arthritis Foundation research grants. BMS was supported by Training Grant: T32AR07080 from the National Institutes of Health, USA.

## Author Disclaimer

The content of this review is the responsibility of the authors and does not necessarily reflect the official views of the funding agencies.

## Conflict of Interest

JH is an Inventor of patents owned by the Regents of the University of Michigan that are licensed to Zydus-Cadila, to whom he is a consultant.

The remaining authors declare that the research was conducted in the absence of any commercial or financial relationships that could be construed as a potential conflict of interest.

## Publisher’s Note

All claims expressed in this article are solely those of the authors and do not necessarily represent those of their affiliated organizations, or those of the publisher, the editors and the reviewers. Any product that may be evaluated in this article, or claim that may be made by its manufacturer, is not guaranteed or endorsed by the publisher.
